# An Open-Source Analysis of Cardiomyopathy Using Machine Learning and Electrocardiograms

**DOI:** 10.3390/diagnostics16050719

**Published:** 2026-02-28

**Authors:** Arda Altintepe, Asu Rustemli, Amir Reza Vazifeh, Jason W. Fleischer

**Affiliations:** 1 Horace Mann School, Bronx, NY 10471, USA; 2 Ocean Cardiovascular, LLC, Toms River, NJ 08755, USA; asurustemli@gmail.com; 3 Department of Electrical and Computer Engineering, Princeton University, Princeton, NJ 08544, USA; av6580@princeton.edu (A.R.V.); jasonf@princeton.edu (J.W.F.); 4 Princeton Precision Health, Princeton University, Princeton, NJ 08544, USA; 5 Omenn-Darling Bioengineering Institute, Princeton University, Bioengineering Building, 35 Ivy Lane, Princeton, NJ 08540, USA

**Keywords:** electrocardiogram (ECG), vectorcardiogram (VCG), machine learning, dilated cardiomyopathy, hypertrophic cardiomyopathy, obstructive hypertrophic cardiomyopathy, ischemic dilated cardiomyopathy

## Abstract

**Background/Objectives:** Dilated cardiomyopathy (DCM) and hypertrophic cardiomyopathy (HCM) are common cardiomyopathies associated with heart failure. Electrocardiogram (ECG) screening before an echocardiogram could help streamline diagnosis, particularly in rural areas. Prior ECG–machine learning (ML) studies do not use open-source data when studying cardiomyopathy, and very few proprietary studies directly compare HCM and DCM or address ECG differences within obstructive (HOCM) and non-obstructive HCM (HNCM). **Methods:** Standard and vectorcardiogram-derived (VCG) ECG features were extracted from the MIMIC-IV-ECG database. The final cohort comprised 599 patients (HCM = 208 [HOCM = 99, HNCM = 53, unknown = 56]; DCM = 391 [ischemic cardiomyopathy with left ventricular dilation = 250, non-ischemic = 141]). Logistic regression (LR) and extreme gradient boosting (XGBoost) with five-fold cross-validation separated HCM from ischemic cardiomyopathy with left ventricular dilation (DCM-I) and non-ischemic DCM (DCM-NI), and HOCM from HNCM. **Results:** Using the area under the receiver-operating-characteristic curve (AUC-ROC) as the performance metric, LR achieved high discrimination of HCM from DCM-I (0.92) and DCM-NI (0.90). However, differentiating HOCM from HNCM proved more difficult (XGBoost = 0.81; LR = 0.75). Both DCM subtypes (especially ischemic) showed lower QRS amplitudes and right-posterior ventricular gradient orientation; HCM displayed higher amplitudes and larger, more complex T-loops. Within HCM, HOCM had stronger leftward electrical activity and more dipolar to non-dipolar QRS energy after singular value decomposition. **Conclusions:** Using only open-access data, we demonstrate an interpretable ECG-based pipeline that discriminates cardiomyopathy and highlights distinct features. While detecting obstruction remains difficult, ECG features provide measurable separation, supporting possible diagnostic screening and offering a reproducible framework for future studies.

## 1. Introduction

Cardiomyopathy (CM) describes a diverse group of diseases affecting the heart muscle [1]. Dilated cardiomyopathy and hypertrophic cardiomyopathy represent the two most common forms of CM with the highest risk of heart failure and sudden cardiac death [1]. In dilated cardiomyopathy (DCM), the ventricular chambers enlarge, and the heart’s pumping ability weakens, reducing stroke volume and impairing systolic function [1,2]. DCM can develop from causes such as genetic mutations, myocarditis, or toxic exposures like alcohol. By contrast, chronic coronary artery disease can lead to ischemic myocardial injury and left ventricular systolic dysfunction, with or without regional wall-motion abnormalities. In some cases, there may be left ventricular dilation, for which those patients could, morphologically, be considered to have ischemic cardiomyopathy with left ventricular dilation (DCM-I). In hypertrophic cardiomyopathy (HCM), the ventricular walls thicken abnormally, often causing impaired diastolic filling and, in some cases, dynamic outflow obstruction [1,2,3,4]. Unlike DCM, HCM usually occurs because of inherited genetic changes in the heart muscle proteins [1,2].

The treatments of DCM and HCM are distinct. Thus, early diagnosis is imperative to determine the correct treatment, halt the progression of DCM, limit symptoms, and improve prognosis [2]. Diagnosis is traditionally done through imaging studies, such as echocardiography and magnetic resonance imaging (MRI) [2]. Currently, the most widely available diagnostic tool is an echocardiogram. However, echocardiograms may not be as practical in rural areas or developing countries due to the high cost or lack of infrastructure and well-trained cardiac sonographers [3].

The electrocardiogram (ECG) may present an inexpensive and noninvasive screening approach before conducting an echocardiogram. Vectorcardiography (VCG) extends this concept by transforming the ECG into a three-dimensional representation of the heart’s electrical activity. Instead of looking at each lead separately, VCG traces the overall direction and magnitude of the instantaneous heart vector in 3D space. This approach can highlight patterns of depolarization and repolarization that standard ECG features may miss, offering additional discriminatory power for cardiomyopathy diagnosis. Here, we leverage machine learning (ML) and VCGs to differentiate HCM, DCM, and several phenotypes.

Studies on artificial intelligence (AI) and machine learning (ML) have recently demonstrated potential in classifying cardiomyopathy [5,6,7,8,9], which could prove useful in diagnosis. However, most studies use echocardiograms. For example, R. Nasimov created a convolutional neural network (CNN) to classify DCM from HCM based on echocardiogram imaging, reaching an accuracy of 96.6% [5]. Zhou et al. present an echo-based XGBoost algorithm with a 0.93 AUC to classify ischemic cardiomyopathy from dilated cardiomyopathy [6]. Machine learning and deep-learning models that identify hypertrophic cardiomyopathy from ECG data also exist; Ko and Attia constructed a deep-learning convolutional neural network to identify HCM from non-HCM patients with an AUC of 0.96 [7]. Kokubo et al. offer deep-learning models with acceptable accuracies to detect left ventricular dilation and left ventricular hypertrophy from ECG waveforms [8]. Yet, left ventricular hypertrophy and left ventricular dilation are only common features of HCM and DCM patients, respectively; their presence alone cannot be used to diagnose CM [9]. A recent study highlighted advanced ECG (A-ECG) features derived from vectorcardiogram (VCG) transformations, which achieve high accuracy in identifying patients with non-ischemic or ischemic cardiomyopathy, apical HCM, and asymmetric septal HCM within a broader cohort comprising healthy individuals and those with other cardiac diseases [10]. Their ischemic and non-ischemic cardiomyopathy groups were defined by reduced left ventricular ejection fraction (LVEF) rather than explicit left ventricular dilation criteria; therefore, these categories may not correspond to DCM (dilation and dysfunction). Their primary focus was apical HCM (and further subtype) detection rather than ECG feature comparisons for HCM vs. DCM.

Approximately one-third of patients with HCM develop dynamic left ventricular outflow tract obstruction (LVOTO). Resting LVOTO is a strong, independent predictor of progression to severe heart-failure symptoms and of sudden cardiac death [4]. Obstructive HCM (HOCM) requires negative inotropic drugs, and patients with HOCM may need invasive therapies such as septal reduction. Identifying LVOTO without ultrasound imaging remains challenging. A 2023 cross-sectional study of 200 HCM patients reported that standard surface ECG variables could not reliably separate HOCM from non-obstructive HCM (HNCM) [11]. This negative result highlights a diagnostic gap that advanced ECG analytics might fill. Guo et al. prospectively developed and externally validated a logistic regression model using only two standard ECG parameters (P-wave interval and SV1) that differentiated HOCM from HNCM with C-statistics of 0.805 in a temporal validation cohort and 0.776 in an external cohort [12]. To our knowledge, apart from a single pragmatic model by Guo et al., no peer-reviewed study has demonstrated meaningful ECG-based discrimination of obstructive versus non-obstructive HCM.

Additionally, no prior study to date has relied on publicly available data for ECG-based cardiomyopathy diagnosis. The use of open-source datasets presents unique challenges, as these datasets are typically not curated for the specific task and often lack reliable labels and standardized data structures. In contrast, reliance on proprietary datasets restricts reproducibility and broad access within the research community. The Medical Information Mart for Intensive Care IV is a large, deidentified dataset of patients admitted to the emergency department or an intensive care unit at the Beth Israel Deaconess Medical Center in Boston, MA [13,14,15]. The dataset is open-access and available on PhysioNet. In this study, we explore the possible applications of using open-source data with advanced feature analysis to quantify the discriminatory ability of ECG within cardiomyopathy and its functional phenotypes. Specifically, we run statistical tests and develop machine learning models to separate hypertrophic cardiomyopathy from both ischemic and non-ischemic dilated cardiomyopathy, and also detect LVOT obstruction within hypertrophic cardiomyopathy.

## 2. Materials and Methods

Section 2 outlines the overall methodological workflow. We first describe cohort identification and label derivation from MIMIC-IV data, then detail ECG and vectorcardiogram (VCG) feature extraction, and finally present the machine learning classifiers and evaluation framework used for phenotype discrimination. No specialized equipment or instruments were used in this study. All analyses were performed on de-identified, publicly available datasets, using software tools described below.

### 2.1. Patient Cohort

All data were obtained from the Medical Information Mart for Intensive Care (MIMIC)-IV (version 3.1), a large deidentified dataset of patients admitted to the emergency department or an intensive care unit between 2008 and 2022 at the Beth Israel Deaconess Medical Center in Boston, MA [13]. Hospital admissions with International Classification of Diseases (ICD) 9 and 10 discharge codes related to dilated cardiomyopathy (DCM), ischemic cardiomyopathy (ICM), and hypertrophic cardiomyopathy (HCM) were mapped to patient IDs and demographic information. ICD codes “I420,” “I426”, “4255”, and “4257” were used for dilated cardiomyopathy, while “I255” was used for ischemic cardiomyopathy, and “I421”, “I422,” “4251,” “42511,” and “42518” were used for hypertrophic cardiomyopathy. For at least one hospital admission, 701 patients were given a DCM code, 1527 were given an ICM code, and 522 were given an HCM code. The MIMIC-IV-ECG (version 1.0) module was then used to map electrocardiograms to patients in the cohort [16]. We only included patients with an ECG taken during their hospital stay of diagnosis, retaining 2003 unique patients, or 73% of the cohort. ECGs with paced rhythms, artifact ECGs, bundle branch blocks, left anterior fascicular or posterior blocks, intraventricular conduction defects, prominent premature ventricular contractions, atrial fibrillation, or atrial flutter were excluded from the study, removing 421 patients.

However, upon manual review of the discharge notes for several corresponding admissions located in the MIMIC-IV-Note module [17], many ICD diagnoses did not match their physician-written discharge reports for the stay. For example, the HCM ICD codes for “obstructive hypertrophic cardiomyopathy” and “other hypertrophic cardiomyopathy” were often misaligned with the determination of whether the LVOT gradient was actually obstructive, as indicated in the discharge notes. Also, from just ICD codes, there is no way to tell whether an ICM-coded patient also has LV dilation. Many patients did not have an echocardiogram taken during their hospital admission, so these stays could not be trusted to provide an accurate label.

To create more reliable diagnosis labels without relying on closed-source expert review, we leveraged a large language model (LLM) to analyze the discharge reports. Specifically, we chose GPT-4.1 for direct text classification [18] as its flexibility handles the heterogeneity of reports far better than conventional rule-based natural language processing pipelines. To ensure reproducibility, temperature was set to 0. In the first call, the LLM was instructed to carefully read a discharge report and assign HCM, dilated LV systolic dysfunction (echocardiogram criteria were reduced LVEF and LV dilation, including ischemic cardiomyopathy with LV dilation), or neither based strictly on current echocardiogram criteria and explicit physician documentation. The model functioned as an information extractor rather than a clinical decision-maker. A numerical left ventricular ejection fraction (LVEF) was also extracted when reported, which could be used for further analyses. Most discharge notes were classified as neither, which included ambiguous cases or those that specified ischemic or alcoholic cardiomyopathy without echocardiogram-supported LV dilation. For admissions labeled HCM, another decision was made to extract clear current-echocardiogram-verified LVOT obstruction (HOCM vs. HNCM vs. unknown), including whether a septal reduction procedure (myectomy or alcohol ablation) occurred during the admission. For admissions labeled as dilated LV systolic dysfunction cases, the model classified ischemic status (ischemic [DCM-I] vs. non-ischemic [DCM-NI] vs. unknown) based on clear documentation of coronary artery disease, myocardial infarction, revascularization, or explicit ischemic diagnostic terms. Cases with unknown statuses were dropped. Morphological HCM phenotypes, including septal and apical variants, were eventually not considered due to the lack of specification in the reports and minimal sample size. For each classification step (both the primary HCM vs. DCM distinction and the subsequent subtype assignments), the model provided direct quotes from the text supporting the final decision.

**Figure 1:**
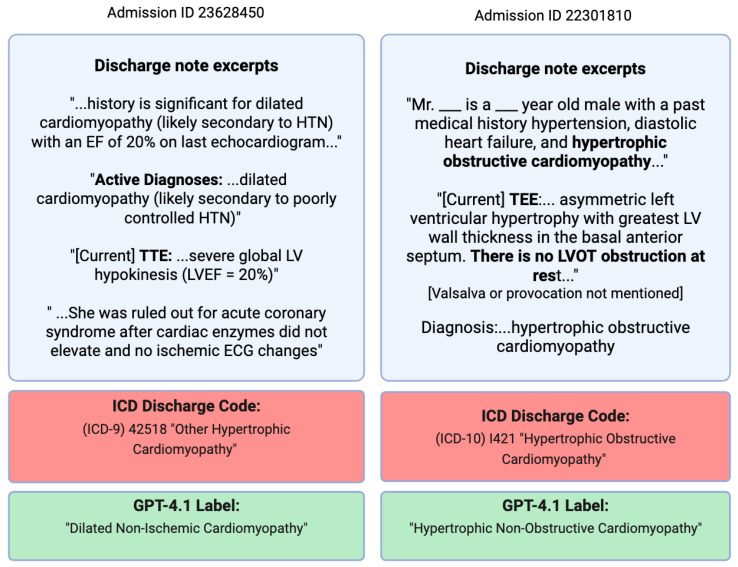
Two discharge notes from unique patients with corresponding ICD codes and final GPT-4.1 labels for their hospital visit. The patient on the (**left**) presents with clear non-ischemic DCM but received an incorrect HCM diagnosis code, which was later corrected by GPT-4.1. On the (**right**), the patient is indicated to have a history of HOCM and received an HOCM diagnosis. However, the echocardiogram findings from the hospital visit directly indicate no LVOT obstruction with no mention of Valsalva obstruction, so the patient should be labeled HNCM. This was correctly done by GPT-4.1, which gives full emphasis to the current echocardiogram.

The derived labels and supporting excerpts were submitted to PhysioNet for dissemination under credentialed access and are currently under review. By design, the model was constrained to assign diagnoses only when they were documented explicitly in the discharge text and supported by extracted excerpts, rather than inferred. As a result, manual review of all 599 (208 HCM, 250 DCM-I, and 141 DCM-NI) final cases, excluding those labeled “neither”, confirmed consistency with the underlying clinical documentation. Two examples of incorrect ICD diagnoses with corrected GPT-4.1-derived diagnoses are shown in Figure 1.

**Figure 2:**
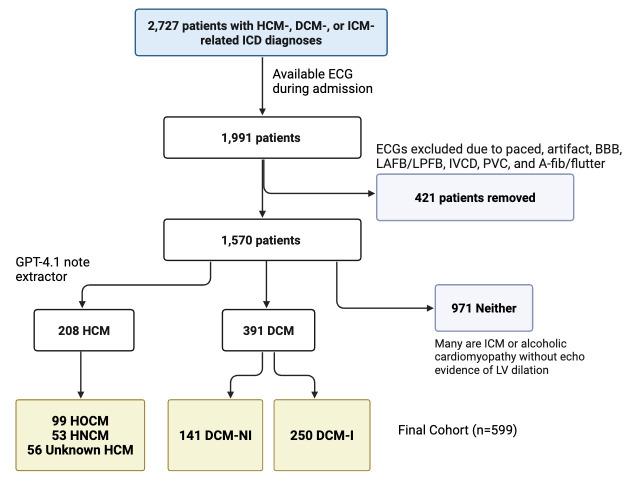
Flow chart showing the study population.

A total of 208 patients with hypertrophic cardiomyopathy (99 obstructive, 53 non-obstructive) and 391 patients with dilated cardiomyopathy (250 ischemic, 141 non-ischemic) remained in the final cohort. One ECG was kept per patient, and priority was given to clean ECGs with a definitive phenotype label (not HCM unknown). Figure 2 shows the full filtering history of the cohort.

### 2.2. ECG Feature Extraction

**Figure 3:**
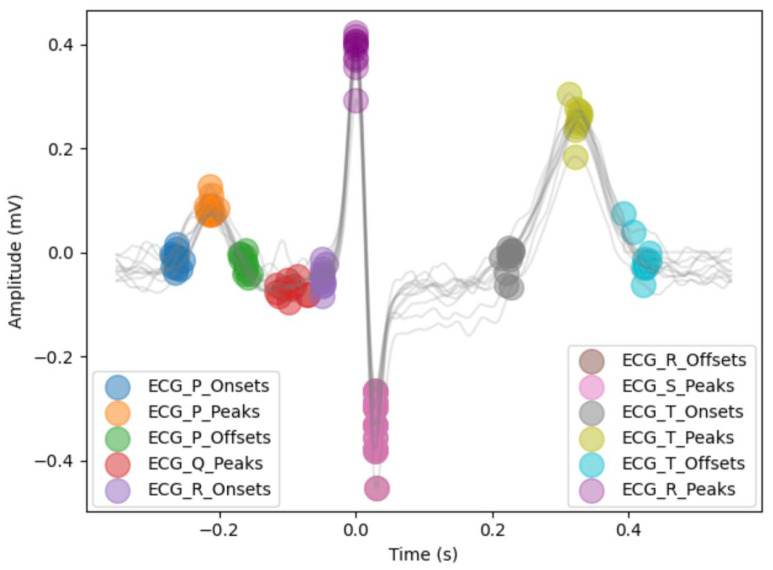
Visual of all detected peaks, onsets, and offsets for a sample lead II in the study population using the “prominence” method. Heartbeats and ECG markers from the 10 s lead II are overlaid. Time (s) is relative to the location of R peaks. The example S-peak and R-offset markers are stacked at the same location. Note that there are no Q-onsets or S-offsets provided in the “prominence” method.

Each ECG wave measures electrical impulses during the stages of a heartbeat. The Waveform Database Software Package (version 10.7.0) was used to convert the waveforms sampled at 500 Hz into a data frame showing time in milliseconds and amplitude for each lead [19]. NeuroKit2 (version 0.2.12), an open-source toolbox for physiological signal processing, was used to clean and determine the locations of most onsets, peaks, and offsets of Q, R, S, T, and P waves for each remaining ECG (illustrated in Figure 3) [20]. The “prominence” method proposed by Emrich et al. was used for delineation [21]. Delineation was done for each of the 12 ECG leads, which represent different electrical views obtained from the anterior chest and limbs.

Median peak amplitudes, as well as intervals and wave durations, were calculated for each wave in reference to the isoelectric PR baseline. ECG waveforms were also transformed using Fourier analysis to extract power-spectral and higher-order statistical moment features for each lead, e.g., the mean frequency in lead V1 (Hz) and the time-domain kurtosis in lead V3. These features were selected to quantify chamber-specific depolarization magnitude, conduction timing, and waveform morphology changes commonly associated with hypertrophy, dilation, and myocardial remodeling.

### 2.3. Vectorcardiogram Feature Extraction

Vectorcardiogram (VCG) features were included because three-dimensional vector analysis can reveal spatial activation and repolarization patterns that are not fully captured by individual surface ECG leads. A VCG representation of an ECG can be built from eight linearly independent leads (I, II, V1, V2, V3, V4, V5, and V6), and having fixed global P, QRS, and T intervals from the standard ECG allows for the construction of P, QRS, and T loops in the VCG projection. Because “prominence” delineation does not locate Q-onsets and S-offsets by default, we developed an automated pipeline using discrete wavelet transform delineation and further amplitude threshold checks to ensure a full QRS-interval selection algorithm. The P-wave, QRS, and T-wave intervals relative to their R-peaks in each of the eight independent leads were combined into a fixed global median. The median was then applied to a mean heartbeat for each lead. The mean heartbeats were calibrated using the R-peak indices of lead V5 to ensure consistent temporal indices throughout all peaks using a reference lead with clear and large R-waves. Kors’ regression matrix transformation projected eight 850 ms mean heartbeats into a 3D VCG space [22]. Using the fixed global P-wave, QRS, and T-wave windows, the analyzed VCG features included peak spatial magnitude, loop geometry, ventricular-activation time, time–voltage area, QRS-T angles, ventricular-gradient strength and direction, and other amplitude or area-based features that describe cardiac depolarization and repolarization.

Finally, all derived ECG features were concatenated with features provided from MIMIC-IV ECG machines: 7 global numerical measurements, including QRS duration and axis, as well as 11 Boolean ECG features, such as “left ventricular hypertrophy”, extracted from the MIMIC-IV machine-generated report text. The total feature set included 14 lead-specific amplitude and Fourier-based features, 30 global interval and axis-based features, 11 Boolean summary features, and 41 VCG-derived features. In addition, 500 electrically normal ECGs from the MIMIC-IV-ECG project underwent the same feature extraction pipeline and were used only for visual comparison (not included in statistical testing or model training).

### 2.4. Machine Learning Model

Two machine learning algorithms were trained to assess the discriminatory ability between hypertrophic and dilated cardiomyopathy phenotypes across three binary classification tasks: HOCM vs. HNCM, HCM vs. DCM-NI, and HCM vs. DCM-I. For HOCM vs. HNCM, only the 15 features with the highest discriminatory ability were chosen as model inputs due to the smaller sample size. The first model trained for each task, multivariate logistic regression (LR), uses a linear combination of predictors, fitted with the liblinear solver (L1 penalty, C = 0.1). Because logistic regression cannot accommodate missing data, we removed the three features with the greatest missingness to preserve sample size and then excluded patients with any remaining missing values, performing complete-case analysis. The second model, extreme gradient boosting (XGBoost), is a non-linear ensemble method that builds decision trees sequentially and re-weights misclassified samples to improve performance. Both models were implemented in scikit-learn (v1.5.1) and trained on the same data. Performance was estimated with consistent 5-fold stratified cross-validation using out-of-fold probabilities. We report the area under the receiver-operating-characteristic curve (AUC-ROC), which summarizes rank discrimination across thresholds, and the area under the precision–recall curve (AUC-PR), which is particularly informative in the setting of class imbalance. We also report sensitivity and specificity calculated at the threshold obtained by maximizing Youden’s J statistic.

## 3. Results

### 3.1. HOCM vs. HNCM: Feature Differences

**Figure 4:**
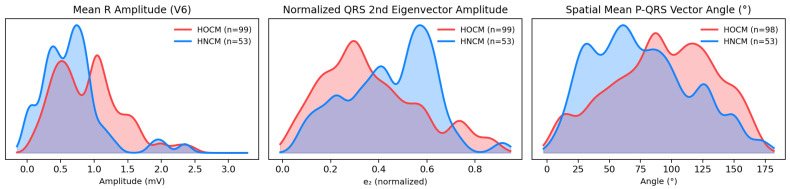
Kernel density estimate (KDE) plots of three ECG-derived features between obstructive and non-obstructive hypertrophic cardiomyopathy. One HOCM patient did not have a valid P-loop detected in the VCG feature extraction.

Obstructive and non-obstructive HCM were compared across all ECG variables. Fourteen representative variables that met the significance threshold (*p* < 0.05) are summarized in Table 1; the full set of variables meeting the *p* < 0.05 threshold is provided in Table S1. In Table 1, variables are listed with median for each group [IQR, interquartile range], the size of their effect (Cliff’s delta or risk difference), and the *p* value (Mann–Whitney U test). Lead names and units are included in the variable labels; the amplitude feature, SaVR, for example, denotes the median absolute value of the S-wave amplitude in lead aVR measured from the prior isoelectric PR baseline. QRS onset and end are global MIMIC-IV numerical measures from a representative heartbeat. Figure 4 shows the kernel density estimates (KDEs) for three ECG features.

#### 3.1.1. Amplitude and Interval Findings

R/S amplitude ratios were consistently higher in HOCM across all left-lateral leads (I, aVL, and V5–V6) and all inferior leads (II, III, and aVF), while HNCM showed higher ratios in the rightward leads (aVR and the right precordials V1–V2) (all *p* < 0.05, Table S1). Because the RS ratio increases with a larger positive R-wave and a smaller negative S-wave, a higher ratio indicates greater net depolarization along that lead’s axis. In lead V6, the median R-amplitude was 0.88 [0.52–1.18] in HOCM and 0.66 [0.35, 0.83] in HNCM (Table 1 and Figure 4). Temporally, in a representative heartbeat starting 40 ms before the representative P-onset, the QRS-end occurred later in non-obstructive HCM patients. The P duration was also longer in HNCM, lasting a median of 120 ms compared to 108 ms in HOCM.

#### 3.1.2. Vectorcardiogram Findings

For the VCG-derived features, HNCM had a higher normalized amplitude for the second QRS eigenvector (the ratio of the second-to-first singular of the QRS-loop [σ_2_/σ_1_]) and the total QRS non-dipolar-to-dipolar ratio ([σ_2_ + σ_3_]/σ_1_) (Figure 4 and Table 1). Thus, in comparison to the dominant axis, the second and third orthogonal axes captured more energy during depolarization, meaning depolarization was less planar or spatially uniform in HNCM. Additionally, the angle between the mean spatial P and QRS vectors was larger in HOCM, while the angle between the first QRS- and T-eigenvector was larger in HNCM (Figure 4).

### 3.2. HCM vs. DCM: Feature Differences

Descriptive baseline characteristics are provided for cohort context in Table 2. Formal demographic comparisons were not performed because the cohort is not intended to be representative, and the primary analyses focus on ECG feature discrimination.

DCM-NI was younger (median: 56 y) than DCM-I (69 y) and HCM (67 y), and both DCM-NI and DCM-I were predominantly male (~81% and 76%) compared with HCM (~46% male). Race distributions varied, with DCM-NI comprising a higher proportion of Black patients (~35%) and DCM-I a higher proportion of White patients (70%).

All amplitude, duration, Fourier, and VCG-derived features were tested for significance within the DCM-NI, DCM-I, and HCM (obstructive, non-obstructive, and unknown together). A total of 500 electrically normal ECGs were also included for visual comparison, but not included in statistical tests. A full table containing all features is available in Table S2.

**Figure 5:**
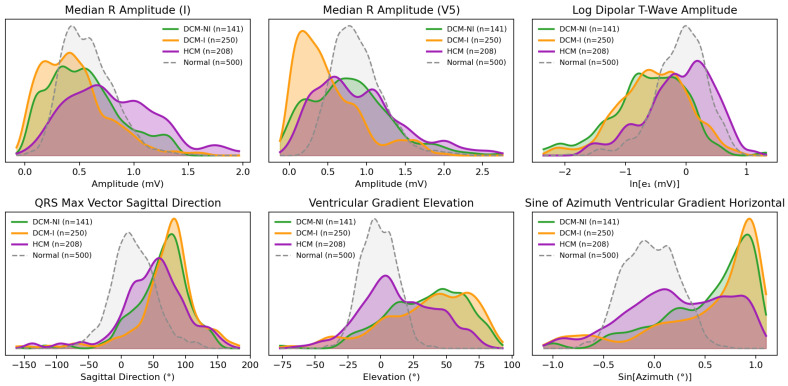
Kernel density estimate (KDE) plots of six ECG-derived features between non-ischemic DCM (DCM-NI), ischemic DCM (DCM-I), hypertrophic cardiomyopathy (HCM), and 500 electrically normal ECGs.

HCM was found to have the highest QRS amplitudes throughout all leads, with particularly large R-wave amplitudes in limb leads (Table S2). For example, the median R-wave amplitude in lead I was 0.76 mV for HCM, 0.51 mV for DCM-NI, 0.41 mV for DCM-I, and 0.55 mV for the electrically normal cohort (Table S2 and Figure 5). Throughout the precordial leads, DCM-I displayed especially low amplitudes: in V5, the median R-wave amplitude for DCM-I was only 0.39 mV, in comparison to 0.76 mV in DCM-NI, 0.80 mV in HCM, and 0.84 mV for normal ECGs. HCM also had the largest maximum amplitude of the spatial QRS vector at 1.15 mV, in comparison to 1.08 mV for DCM-NI, 0.86 mV for normal ECGs, and 0.85 mV for DCM-I. DCM-I consistently showed time-domain skewness closer to zero and lower kurtosis for most leads, indicating lower overall amplitudes, broader durations, and more symmetric waveforms. T-waves were flatter in both DCM-I and DCM-NI (low spatial maximum-to-mean vector ratio).

As referenced in Table S2, the QRS duration was longest in DCM-I (107 ms vs. 86 ms in normal ECGs). While also having longer QRS durations, non-ischemic dilated cardiomyopathy displayed shortened repolarization durations, primarily in the terminal phase. The median T-wave duration in DCM-NI was around 35 ms shorter than normal, with the downslope alone being over 30 ms shorter. While the duration was not corrected for the shorter RR interval (median 681 ms), the upslope of the T-wave was still over twice as long as the downslope (T-index 2.24) in DCM-NI compared to a ratio of only 1.13 and 1.27 for normal ECGs and HCM. In total, 27% of DCM-NI patients had sinus tachycardia rhythms compared to only around 12% of both HCM and DCM-I.

**Figure 6:**
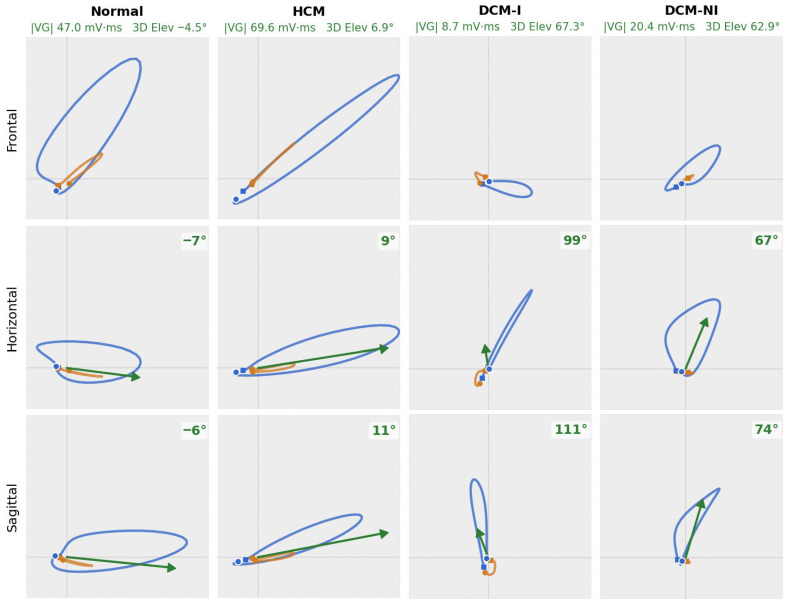
Representative vectorcardiograms for normal, HCM, DCM-I, and DCM-NI patients. The QRS loop is in blue, and the T-loop is in orange. The ventricular gradient (VG) vector is marked in green for the horizontal and sagittal projections; it was not featured for the frontal view, as the standard QRS and T axes from the frontal view were not significant. The length of the VG vector is the magnitude of the projection on the shown view. The HCM patient shows larger loops, with slightly larger VG azimuths in comparison to the normal patient. Both DCM patients show loops smaller in amplitude in all projections, accompanied by small amplitude VG vectors with far higher 3D elevation and higher azimuth angles within the horizontal and sagittal projections.

Compared with dilated cardiomyopathy (ischemic and non-ischemic) and electrically normal ECGs, hypertrophic cardiomyopathy showed both greater T-loop energy and spatial complexity. The first T-loop singular value (log dipolar T-wave amplitude) was largest in HCM (median −0.01) vs. DCM-NI (−0.58) and DCM-I (−0.53), with normal ECGs near −0.04 (Table S2 and Figure 5). The second T-loop singular value, a marker of non-planarity, was also larger in HCM (−1.39) than in DCM-NI (−1.75) and DCM-I (−1.67), with normal ECGs at −1.77 (Table S2). Multiple directional VCG metrics highlighted distinct abnormalities in dilated cardiomyopathy (both ischemic and non-ischemic), showing very similar right-posterior orientation shifts, while HCM remained closer to normal. For the ventricular gradient (VG)—the spatial integral of the instantaneous heart vector over depolarization and repolarization—the median sine of the azimuth angle in the sagittal plane was 0.86 (DCM-NI) and 0.87 (DCM-I) versus 0.31 (HCM) and −0.06 (normal ECGs); the horizontal projection showed a similar separation (0.68 and 0.77 vs. 0.23 and −0.04). 3D VG elevation (from the XY plane) differed by only ~3° between DCM-NI and DCM-I but by around ~30° relative to HCM and ~40° relative to normal ECGs (see Table S2 for full statistics). Figure 6 shows vectorcardiogram appearances of a representative patient from each group.

### 3.3. Classifier Performance

**Figure 7:**
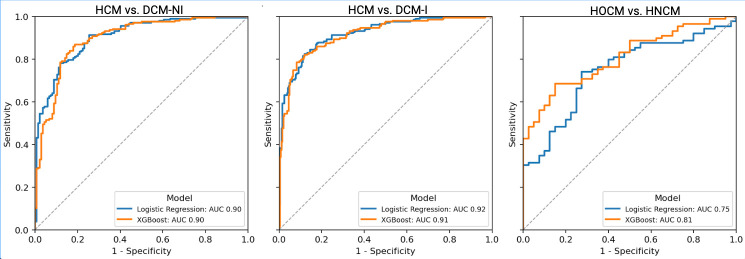
Pooled out-of-fold receiver operating characteristic (ROC) curves for each task and model type in the internal validation cohort. Each ROC curve plots sensitivity (true positive rate) against 1 −specificity (false positive rate). AUC is the area under the curve. The dashed diagonal line represents the performance of a random classifier (AUC = 0.5).

For HCM and DCM classification, multivariate logistic regression and XGBoost models were trained on the complete feature set. Due to a lower sample size, only 15 representative ECG features (nine amplitude, one duration, and five VCG) were input into the LR and XGBoost for HOCM vs. HNCM. Performance metrics are displayed in Table 3. The receiver operating characteristic (ROC) curves are shown in Figure 7. After applying missing-data handling, the final analytic sample sizes for machine learning were as follows: HCM vs. DCM-I (*n* = 448 of 458 available), HCM vs. DCM-NI (*n* = 340 of 349), and HOCM vs. HNCM (*n* = 129 of 152). For comparability, both logistic regression and XGBoost were trained and evaluated on identical analytic subsets for each task.

## 4. Discussion

In our study, classifiers built solely from electrocardiogram inputs were developed to distinguish hypertrophic cardiomyopathy and dilated cardiomyopathy. Some conditions were easier to distinguish, including HCM as a whole versus ischemic or non-ischemic dilated cardiomyopathy (AUC-ROC: 0.92 and 0.90). Others, such as obstruction with ECG, were more challenging (AUC-ROC: 0.81 with XGBoost). The ECG features tested included a combination of lead-specific amplitude and Fourier-based features, global interval/duration features, and vectorcardiogram-derived spatial features.

### 4.1. General Cardiomyopathy Analysis

Demographically, the median age of non-ischemic DCM (DCM-NI) patients was over 11 years younger than patients with DCM-I and HCM, while both ischemic and non-ischemic DCM were near 80% male, in comparison to ~46% of HCM patients. These demographics align with the EuroHeart Failure Survey II, which also found that DCM was more prevalent in males than females [23]. QRS amplitudes were consistently low in DCM, findings frequently observed in non-ischemic DCM and often linked to myocardial fibrosis on cardiac MRI [24]. In ischemic DCM, precordial R-wave amplitudes were even lower, consistent with the loss of anterior forces from infarct scarring [25,26]. Uncorrected (rate-dependent) repolarization durations were shorter in DCM-NI. These patients also showed higher heart rates, with more than twice the prevalence of sinus tachycardia compared with DCM-I and HCM. The T-index (upstroke/downstroke) was also higher in DCM-NI. Using a prominence-based delineator, the T-end is anchored to the right-hand base of the T peak (the adjacent minimum on the descending limb). When the TP interval shortens or the baseline rises (due to more frequent sinus tachycardia and a lower median RR interval), the upcoming P-wave elevates that minimum and shifts the base earlier, possibly shortening the T-wave downstroke. We therefore interpret the T-index cautiously; this is unlikely to affect VCG orientation results, which depend on the higher-amplitude mid-T portion rather than the terminal tail.

VCG geometry revealed that both ischemic and non-ischemic DCM exhibited right-posterior orientation shifts of the ventricular gradient (VG) and more negative T-vector angles. HCM preserved a more leftward VG but demonstrated larger and more complex and spatially non-planar T-loops. The similarity between non-ischemic and ischemic DCM in these VCG angles suggests that global ventricular dilatation and conduction delay, rather than the presence of infarct scarring, dominate the three-dimensional orientation of electrical activation in dilated cardiomyopathy.

### 4.2. HCM Subtype Analysis

While harder to differentiate on ECG, several distinctions were found between obstructive and non-obstructive HCM. ECG-based discrimination between HOCM and HNCM is most relevant as a screening tool when imaging is not immediately available, helping identify patients who may warrant earlier LVOT gradient assessment. Because obstruction is dynamic and load-dependent, a resting 10 s ECG may reflect remodeling associated with obstruction rather than the instantaneous gradient. Through lead-amplitude analysis, HOCM consistently displayed more positive electrical activity on the left leads, while HNCM displayed the opposite. The prominent left-leaning electrical activity may be originating from hypertrophy severe enough to cause the obstructive physiology, which may explain the amplified electrical signals coming from the left side of the myocardium.

At the same time, left ventricular hypertrophy alone was suggested by Savage et al. to be insufficient in determining the LVOT gradient [27]. Indeed, we note that in our cohort, the presence of left ventricular hypertrophy was not statistically significant (*p* = 0.59) and only differed ~5% between HOCM and HNCM. This also suggests another reason for the stronger left-lateral depolarization observed in HOCM. Guo et al. found the same overall correlation, with HOCM having larger R-wave amplitudes in left leads and larger S-wave amplitudes in right leads [12]. They used the amplitude of the S-wave in lead V1 (SV1) as one of two variables in a logistic regression model classifying HOCM, which we also found to be larger in HOCM patients. However, the duration of the P-wave—the other variable used in their model—in our cohort was longer in HNCM patients, while their study indicates the opposite effect. No prior study has used VCG-derived features to discriminate HOCM from HNCM. We found HNCM to have larger non-dipolar QRS components in comparison to dipolar components, suggesting that the depolarizations are less planar and spatially more dispersed than in HOCM. Further research is needed here.

### 4.3. Open-Source Data and Limitations

To the best of our knowledge, all studies involved in any form of ECG cardiomyopathy diagnosis utilize non-public proprietary data, and thus, it can be increasingly difficult for other researchers without access to reproduce or create their own findings. This study uses only publicly available MIMIC-IV data and provides a reproducible framework for the use of open-source hospital data and signal-processing with machine learning. We also provide clinically interpretable features in the VCG analysis that link to cardiac anatomy.

Only inpatient data were included from the single-center Beth Israel Deaconess Medical Center. Inclusion of outpatient data may improve results. HOCM was more prevalent than HNCM in our cohort, likely due to echocardiogram-verified LVOTO being more common in an inpatient setting. With the use of the MIMIC-IV database, all data rely on the accuracy and quality of the project’s diagnostic labels. This includes the validity of MIMIC’s machine-generated ECG reports, which were used to exclude patients with paced rhythms and identify patients with atrial fibrillation or other categorical features.

The accuracy of the diagnosis was confirmed by a large language model, and while the model extracted reliable results visibly (which are also public), large AI models can still hallucinate on occasion. Research with open-source data is confined to the information provided in the project, and many reliable HCM and DCM phenotype labels were not available. We could not include morphological HCM phenotypes, such as septal or apical HCM, into our analysis due to limited, reliable sample sizes. Likewise, we did not have enough information to sub-classify ischemic dilated cardiomyopathy either. With further labels, one can implement the provided workflow to differentiate diastolic dysfunction physiology in HCM from DCM or other relevant phenotype combinations.

## 5. Conclusions

We provide the first framework to explore different cardiomyopathy presentations using open-source electrocardiograms. Our analysis combined standard ECG amplitude and duration features with more advanced signal processing and spatial vectorcardiography. We provide statistically significant features in detecting obstruction in hypertrophic cardiomyopathy, such as higher positive amplitudes in left-facing leads, along with more planar spatial depolarization. We confirm findings from prior studies, which conclude lower QRS amplitudes and higher QRS durations in DCM-NI and DCM-I patients. HCM displays spatially larger and more complex T-waves, while DCM-NI and DCM-I share abnormal QRS- and T-vector angles pointing towards the right ventricle. Finally, we developed an XGBoost model with an AUC-ROC of 0.81 to detect obstruction in HCM, and logistic regression models with an AUC-ROC of 0.92 and 0.90 distinguishing HCM from ischemic and non-ischemic DCM.

## Figures and Tables

**Table 1:** Fourteen ECG variables that differed significantly (*p* < 0.05) between hypertrophic obstructive and non-obstructive phenotypes.

ECG Feature	HOCM (*n* = 99)	HNCM (*n* = 53)	Effect Size	*p*
SV1 (mV)	0.86 [0.60, 1.27]	0.66 [0.31, 0.94]	0.283	0.004
SII (mV)	0.07 [0.03, 0.20]	0.15 [0.06, 0.30]	−0.299	0.002
RV2 (mV)	0.18 [0.08, 0.36]	0.29 [0.13, 0.62]	−0.284	0.004
RV6 (mV)	0.88 [0.52, 1.18]	0.66 [0.35, 0.83]	0.316	0.001
R/S amplitude ratio (II)	10.87 [2.70, 22.02]	2.63 [1.20, 7.27]	0.357	<0.001
R/S amplitude ratio (aVR)	0.18 [0.08, 1.09]	1.04 [0.29, 1.54]	−0.333	<0.001
QRS end (ms)	296.00 [274.00, 325.00]	326.00 [292.50, 359.50]	−0.309	0.002
P duration (ms)	108.00 [98.50, 119.50]	120.00 [102.00, 128.50]	−0.286	0.018
Time-domain skewness (V1)	−2.97 [−3.42, −2.48]	−2.62 [−3.24, −1.08]	−0.290	0.004
R amplitude in lead Y (mV)	0.35 [0.15, 0.66]	0.19 [−0.08, 0.40]	0.289	0.003
Spatial P–QRS mean-vector angle (°)	91.58 [65.38, 124.25]	68.89 [48.25, 100.51]	−0.246	0.012
QRS–T eigenvector-1 angle (°)	42.68 [15.49, 122.53]	67.35 [25.87, 153.79]	−0.228	0.025
Normalized QRS eigenvector-2	0.32 [0.21, 0.49]	0.44 [0.31, 0.59]	−0.257	0.009
ln QRS non-dipolar-to-dipolar ratio	−1.26 [−1.61, −1.02]	−1.04 [−1.33, −0.91]	−0.272	0.006

**Table 2:** Demographic characteristics by cardiomyopathy group. Age is expressed as median [IQR]. Sex and race are shown as percentages (frequency).

Feature	DCM-NI (*n* = 141)	DCM-I (*n* = 250)	HCM (*n* = 208)
Age (years)	56.00 [47.00, 64.00]	69.00 [60.00, 78.00]	67.00 [57.00, 79.25]
Male	80.9% (114)	76.0% (190)	46.2% (96)
Black	34.8% (49)	9.6% (24)	17.3% (36)
Hispanic	6.4% (9)	5.2% (13)	4.8% (10)
White	44.0% (62)	70.0% (175)	63.0% (131)

**Table 3:** Performance summary for binary ECG-based classifiers. Metrics are reported as mean across 5-fold cross-validation. Sensitivity and specificity are calculated at the Youden’s J statistic threshold.

Task	AUC-ROC	AUC-PR	Sensitivity	Specificity
Logistic Regression				
HCM vs. DCM-NI	0.90	0.93	0.78	0.88
HCM vs. DCM-I	0.92	0.92	0.83	0.88
HOCM vs. HNCM	0.75	0.88	0.74	0.73
XGBoost				
HCM vs. DCM-NI	0.90	0.92	0.87	0.81
HCM vs. DCM-I	0.91	0.90	0.79	0.92
HOCM vs. HNCM	0.81	0.92	0.69	0.85

## Data Availability

The code and scripts used to generate the results of this study are openly available at the GitHub repository: https://github.com/againh3x/ECGcardiomyopathy(accessed on 23 February 2026). The data used are within the open-source MIMIC-IV, MIMIC-IV-ECG, and MIMIC-IV-Note modules on PhysioNet. Because access to MIMIC-IV is restricted under a data-use agreement that protects patient privacy, raw or pre-processed patient-level data cannot be redistributed in the attached GitHub repository. Interested researchers can obtain the same data free of charge by completing the data-use certification on PhysioNet. MIMIC-IV-ECG is fully open-access. The project containing GPT-4.1 diagnosis labels corresponding to the admission ID and relevant quotes is currently under review for credentialed access.

## Supplementary Materials

The following supporting information can be downloaded at: https://www.mdpi.com/article/10.3390/diagnostics16050719/s1, Table S1: ECG variables that differ significantly (*p* < 0.05) between obstructive and non-obstructive HCM. Table S2: Descriptive statistics for all ECG features across HCM and DCM groups.

## Abbreviations

The following abbreviations are used in this manuscript:

A-ECG	Advanced electrocardiogram
AUC	Area under the curve
AUC-ROC	Area under the receiver-operating-characteristic curve
AUC-PR	Area under the precision–recall curve
CM	Cardiomyopathy
DCM	Dilated cardiomyopathy
DCM-I	Dilated ischemic cardiomyopathy
DCM-NI	Dilated non-ischemic cardiomyopathy
ECG	Electrocardiogram
HCM	Hypertrophic cardiomyopathy
HNCM	Hypertrophic non-obstructive cardiomyopathy
HOCM	Hypertrophic obstructive cardiomyopathy
ICD	International Classification of Diseases
ICM	Ischemic cardiomyopathy
IQR	Interquartile range
LR	Logistic regression
LV	Left ventricle
LVOTO	Left ventricular outflow tract obstruction
ML	Machine learning
VCG	Vectorcardiogram
VG	Ventricular gradient
WFDB	Waveform Database Software Package
