# Supplementary material for: An Open-Source Analysis of Cardiomyopathy Using Machine Learning and Electrocardiograms
Source: Diagnostics (Basel). 2026 Feb 28;16(5):719. doi: 10.3390/diagnostics16050719 (PMC12984491; doi:10.3390/diagnostics16050719)
Supplement: Supplementary file 1 [file diagnostics-16-00719-s001.zip › diagnostics-4138762-supplementary.pdf]

Table S1. *ECG variables that differ significantly (p < 0.05) between hypertrophic obstructive cardiomyopathy (HOCM) and hypertrophic non-obstructive cardiomyopathy (HNCM). Continuous features are reported as median [IQR], and categorical features as frequency (%). Features are sorted in ascending order of p-value.*

ECG Feature	HOCM (n=99)	HNCM (n=53)	Effect size	*P*
RS ratio II	10.87 [2.70, 22.02]	2.63 [1.20, 7.27]	0.357	<.001
ST slope aVR (mV/ms)	0.01 [0.00, 0.01]	0.00 [0.00, 0.01]	0.342	<.001
RS slope (aVR)	-0.02 [-0.04, -0.01]	-0.01 [-0.03, 0.00]	-0.341	<.001
RS ratio aVR	0.18 [0.08, 1.09]	1.04 [0.29, 1.54]	-0.333	<.001
RV6 (mV)	0.88 [0.52, 1.18]	0.66 [0.35, 0.83]	0.316	.001
QRS end (ms)	296.00 [274.00, 325.00]	326.00 [292.50, 359.50]	-0.309	.002
RS ratio V6	11.96 [6.05, 25.10]	6.03 [1.40, 16.10]	0.307	.002
SII (mV)	0.07 [0.03, 0.20]	0.15 [0.06, 0.30]	-0.299	.002
QRS area V1 (mV·ms)	-0.01 [-0.02, -0.01]	-0.01 [-0.01, 0.00]	-0.299	.002
TR ratio V2	1.65 [0.58, 4.86]	0.79 [0.37, 2.53]	0.293	.003
R-wave amplitude (Lead Y) (mV)	0.35 [0.15, 0.66]	0.19 [-0.08, 0.40]	0.289	.003
Time-domain skewness (V1)	-2.97 [-3.42, -2.48]	-2.62 [-3.24, -1.08]	-0.286	.004
ST slope II (mV/ms)	0.00 [0.00, 0.00]	0.00 [0.00, 0.01]	-0.284	.004
RV2 (mV)	0.18 [0.08, 0.36]	0.29 [0.13, 0.62]	-0.284	.004
SV1 (mV)	0.86 [0.60, 1.27]	0.66 [0.31, 0.94]	0.283	.004
SaVL (mV)	0.04 [0.02, 0.09]	0.09 [0.04, 0.15]	-0.278	.005
QRS area V6 (mV·ms)	0.02 [0.01, 0.03]	0.01 [0.01, 0.02]	0.275	.005
QaVL (mV)	0.01 [0.01, 0.03]	0.02 [0.01, 0.07]	-0.274	.005
ST slope V1 (mV/ms)	0.01 [0.01, 0.02]	0.01 [0.00, 0.01]	0.273	.006
RS ratio V1	0.11 [0.05, 0.23]	0.23 [0.10, 0.55]	-0.273	.006
ln QRS non-dipolar to dipolar ratio	-1.26 [-1.61, -1.02]	-1.04 [-1.33, -0.91]	-0.272	.006
QRS area II (mV·ms)	0.01 [0.00, 0.02]	0.01 [0.00, 0.01]	0.266	.007
SaVR (mV)	0.57 [0.33, 0.87]	0.39 [0.13, 0.59]	0.000	
Time-domain skewness (aVF)	1.06 [-0.97, 2.26]	-0.67 [-1.81, 1.00]	0.258	.009
Normalized QRS eigenvector-2 amplitude	0.32 [0.21, 0.49]	0.44 [0.31, 0.59]	-0.257	.009

RaVR (mV)	0.10 [0.06, 0.23]	0.26 [0.08, 0.55]	-0.253	.010
TR ratio V1	2.14 [0.89, 4.19]	1.26 [0.64, 2.39]	0.000	
RS ratio V2	0.29 [0.06, 0.60]	0.42 [0.22, 1.16]	-0.249	.012
RS ratio I	15.54 [8.74, 27.78]	10.32 [4.99, 18.74]	0.246	.013
Spatial mean P–QRS angle (°)	91.58 [65.38, 124.25]	68.89 [48.25, 100.51]	0.000	
QRS area V2 (mV·ms)	-0.01 [-0.02, 0.00]	-0.01 [-0.02, 0.00]	-0.245	.013
TR ratio aVR	1.45 [0.69, 2.79]	0.77 [0.47, 1.71]	0.245	.013
ST slope V6 (mV/ms)	0.00 [0.00, 0.00]	0.00 [0.00, 0.00]	-0.243	.014
LAD	13/79 (16.5%)	16/53 (30.2%)	-0.171	.016
TIII (mV)	0.11 [0.06, 0.22]	0.18 [0.10, 0.28]	-0.235	.017
P duration (ms)	108.00 [98.50, 119.50]	120.00 [102.00, 128.50]	0.000	
P end (ms)	148.00 [138.50, 159.50]	160.00 [142.00, 168.50]	-0.261	.018
SaVF (mV)	0.10 [0.03, 0.38]	0.21 [0.06, 0.52]	-0.232	.019
Time-domain skewness (II)	2.09 [0.77, 3.06]	1.24 [-0.05, 2.44]	0.228	.021
QRS area aVR (mV·ms)	-0.01 [-0.01, 0.00]	0.00 [-0.01, 0.00]	-0.227	.021
RS ratio aVF	3.92 [0.51, 13.21]	0.89 [0.38, 4.14]	0.224	.023
QRS–T principal eigenvector angle (°)	42.68 [15.49, 122.53]	67.35 [25.87, 153.79]	-0.222	.025
QI (mV)	0.01 [0.01, 0.03]	0.02 [0.01, 0.04]	-0.214	.030
RS ratio aVL	14.47 [6.36, 29.39]	7.41 [1.93, 21.18]	0.212	.032
RV3 (mV)	0.34 [0.12, 0.62]	0.45 [0.21, 0.93]	-0.209	.034
RV5 (mV)	0.95 [0.55, 1.24]	0.68 [0.47, 1.03]	0.208	.035
QRS duration (ms)	90.00 [84.00, 101.00]	96.00 [88.00, 104.00]	-0.207	.036
RS ratio V5	7.00 [1.92, 19.50]	3.05 [1.62, 11.67]	0.000	
QRS onset (ms)	202.00 [187.00, 232.00]	212.00 [197.00, 259.50]	0.000	
Time-domain skewness (V6)	2.91 [2.01, 3.57]	2.62 [0.86, 3.33]	0.201	.042
Direction of max QRS (frontal, °)	25.97 [13.15, 35.43]	18.92 [-8.74, 31.23]	0.200	.042
SV6 (mV)	0.07 [0.04, 0.13]	0.13 [0.04, 0.23]	-0.198	.044

Table S2. *Median [IQR] values of ECG-derived amplitude, interval, spectral and vectorcardiographic features in non-ischemic dilated cardiomyopathy, ischemic cardiomyopathy, hypertrophic cardiomyopathy and 500 electrically normal ECGs (sample sizes shown in column headers). Q-values are Benjamini–Hochberg–adjusted p-values from Kruskal–Wallis tests across the three cardiomyopathy groups (controls excluded), with features sorted in ascending order of q-value.*

Feature	DCM-NI (n=141)	DCM-I (n=250)	HCM (n=208)	Normal ECG (n=500)	q
**RI (mV)**	0.51 [0.30, 0.74]	0.41 [0.22, 0.60]	0.76 [0.50, 1.10]	0.55 [0.41, 0.72]	<.001
**RII (mV)**	0.34 [0.18, 0.61]	0.25 [0.14, 0.44]	0.54 [0.32, 0.92]	0.58 [0.43, 0.78]	<.001
**RIII (mV)**	0.13 [0.04, 0.26]	0.17 [0.07, 0.39]	0.19 [0.07, 0.39]	0.18 [0.07, 0.38]	0.004
**RaVR (mV)**	0.18 [0.09, 0.32]	0.14 [0.07, 0.29]	0.12 [0.06, 0.35]	0.13 [0.07, 0.37]	0.141
**RaVL (mV)**	0.36 [0.20, 0.61]	0.35 [0.19, 0.64]	0.54 [0.31, 0.88]	0.28 [0.13, 0.45]	<.001
**RaVF (mV)**	0.22 [0.09, 0.42]	0.17 [0.07, 0.38]	0.30 [0.12, 0.53]	0.31 [0.17, 0.54]	<.001
**RV1 (mV)**	0.11 [0.06, 0.16]	0.09 [0.04, 0.19]	0.10 [0.05, 0.20]	0.10 [0.07, 0.15]	0.427
**RV2 (mV)**	0.13 [0.07, 0.21]	0.10 [0.04, 0.22]	0.22 [0.08, 0.38]	0.25 [0.14, 0.38]	<.001
**RV3 (mV)**	0.21 [0.08, 0.40]	0.13 [0.04, 0.34]	0.36 [0.17, 0.75]	0.48 [0.32, 0.73]	<.001
**RV4 (mV)**	0.45 [0.19, 0.81]	0.24 [0.08, 0.60]	0.71 [0.34, 1.10]	0.82 [0.59, 1.11]	<.001
**RV5 (mV)**	0.76 [0.43, 1.10]	0.39 [0.18, 0.72]	0.80 [0.48, 1.19]	0.84 [0.65, 1.07]	<.001
**RV6 (mV)**	0.71 [0.43, 1.00]	0.42 [0.22, 0.69]	0.74 [0.46, 1.11]	0.73 [0.55, 0.93]	<.001
**SI (mV)**	0.04 [0.02, 0.08]	0.05 [0.02, 0.11]	0.06 [0.03, 0.12]	0.02 [0.01, 0.05]	0.004
**SII (mV)**	0.08 [0.03, 0.20]	0.08 [0.04, 0.24]	0.08 [0.03, 0.22]	0.03 [0.01, 0.06]	0.517
**SIII (mV)**	0.25 [0.04, 0.54]	0.19 [0.04, 0.59]	0.27 [0.06, 0.76]	0.04 [0.02, 0.21]	0.151
**SaVR (mV)**	0.30 [0.15, 0.52]	0.19 [0.07, 0.36]	0.51 [0.27, 0.77]	0.44 [0.29, 0.60]	<.001
**SaVL (mV)**	0.04 [0.02, 0.12]	0.06 [0.03, 0.15]	0.05 [0.03, 0.11]	0.03 [0.01, 0.11]	0.015
**SaVF (mV)**	0.11 [0.03, 0.34]	0.13 [0.03, 0.36]	0.11 [0.04, 0.42]	0.03 [0.02, 0.08]	0.282
**SV1 (mV)**	0.70 [0.48, 1.01]	0.52 [0.25, 0.91]	0.76 [0.46, 1.09]	0.58 [0.41, 0.76]	<.001
**SV2 (mV)**	1.12 [0.63, 1.50]	0.91 [0.51, 1.40]	0.80 [0.37, 1.36]	0.67 [0.48, 0.94]	0.005
**SV3 (mV)**	0.91 [0.47, 1.53]	0.97 [0.60, 1.50]	0.75 [0.38, 1.23]	0.41 [0.23, 0.63]	0.003
**SV4 (mV)**	0.43 [0.19, 0.81]	0.46 [0.21, 0.85]	0.39 [0.15, 0.77]	0.17 [0.06, 0.31]	0.337
**SV5 (mV)**	0.17 [0.07, 0.35]	0.16 [0.06, 0.46]	0.18 [0.05, 0.37]	0.05 [0.02, 0.14]	0.781
**SV6 (mV)**	0.07 [0.04, 0.17]	0.07 [0.04, 0.21]	0.08 [0.04, 0.17]	0.02 [0.01, 0.05]	0.840
**QI (mV)**	0.01 [0.01, 0.02]	0.01 [0.00, 0.02]	0.02 [0.01, 0.04]	0.01 [0.01, 0.02]	<.001
**QII (mV)**	0.01 [0.01, 0.02]	0.01 [0.01, 0.03]	0.02 [0.01, 0.03]	0.01 [0.01, 0.02]	0.006
**QIII (mV)**	0.02 [0.01, 0.05]	0.02 [0.01, 0.09]	0.02 [0.01, 0.09]	0.02 [0.01, 0.05]	0.465

**QaVR (mV)**	0.06 [0.02, 0.12]	0.04 [0.02, 0.08]	0.05 [0.02, 0.12]	0.08 [0.04, 0.16]	0.001
**QaVL (mV)**	0.01 [0.01, 0.03]	0.01 [0.01, 0.03]	0.01 [0.01, 0.04]	0.01 [0.01, 0.02]	0.137
**QaVF (mV)**	0.01 [0.01, 0.02]	0.02 [0.01, 0.05]	0.01 [0.01, 0.03]	0.01 [0.01, 0.02]	0.164
**QV1 (mV)**	0.01 [0.01, 0.03]	0.01 [0.01, 0.03]	0.02 [0.01, 0.05]	0.01 [0.01, 0.03]	0.041
**QV2 (mV)**	0.01 [0.00, 0.02]	0.01 [0.00, 0.02]	0.01 [0.01, 0.02]	0.01 [0.01, 0.01]	0.085
**QV3 (mV)**	0.01 [0.01, 0.02]	0.01 [0.00, 0.02]	0.01 [0.01, 0.02]	0.01 [0.00, 0.01]	0.185
**QV4 (mV)**	0.01 [0.01, 0.02]	0.01 [0.01, 0.02]	0.01 [0.01, 0.02]	0.01 [0.00, 0.01]	0.434
**QV5 (mV)**	0.01 [0.01, 0.02]	0.01 [0.01, 0.02]	0.01 [0.01, 0.03]	0.01 [0.01, 0.02]	0.021
**QV6 (mV)**	0.01 [0.00, 0.02]	0.01 [0.00, 0.02]	0.01 [0.01, 0.03]	0.01 [0.01, 0.02]	0.055
**TI (mV)**	0.07 [0.04, 0.11]	0.08 [0.04, 0.13]	0.14 [0.08, 0.24]	0.20 [0.14, 0.26]	<.001
**TII (mV)**	0.10 [0.06, 0.16]	0.10 [0.05, 0.15]	0.16 [0.10, 0.29]	0.23 [0.18, 0.29]	<.001
**TIII (mV)**	0.09 [0.04, 0.15]	0.12 [0.06, 0.20]	0.13 [0.07, 0.24]	0.07 [0.04, 0.12]	<.001
**TaVR (mV)**	0.13 [0.06, 0.21]	0.09 [0.05, 0.17]	0.16 [0.10, 0.28]	0.17 [0.11, 0.23]	<.001
**TaVL (mV)**	0.05 [0.03, 0.09]	0.07 [0.04, 0.14]	0.10 [0.06, 0.17]	0.08 [0.04, 0.13]	<.001
**TaVF (mV)**	0.09 [0.05, 0.15]	0.09 [0.05, 0.15]	0.12 [0.07, 0.24]	0.12 [0.08, 0.18]	<.001
**TV1 (mV)**	0.16 [0.10, 0.23]	0.16 [0.09, 0.26]	0.18 [0.10, 0.29]	0.09 [0.05, 0.14]	0.037
**TV2 (mV)**	0.21 [0.14, 0.35]	0.25 [0.14, 0.39]	0.31 [0.15, 0.51]	0.30 [0.18, 0.46]	0.006
**TV3 (mV)**	0.24 [0.14, 0.41]	0.26 [0.15, 0.43]	0.33 [0.18, 0.54]	0.32 [0.21, 0.47]	0.012
**TV4 (mV)**	0.19 [0.09, 0.30]	0.17 [0.09, 0.31]	0.28 [0.15, 0.44]	0.30 [0.21, 0.42]	<.001
**TV5 (mV)**	0.13 [0.07, 0.23]	0.11 [0.06, 0.19]	0.21 [0.12, 0.35]	0.26 [0.18, 0.34]	<.001
**TV6 (mV)**	0.10 [0.06, 0.17]	0.08 [0.05, 0.15]	0.18 [0.09, 0.28]	0.21 [0.15, 0.28]	<.001
**PI (mV)**	0.08 [0.05, 0.10]	0.06 [0.04, 0.09]	0.09 [0.06, 0.11]	0.08 [0.07, 0.10]	<.001
**PII (mV)**	0.12 [0.07, 0.17]	0.09 [0.06, 0.13]	0.11 [0.08, 0.16]	0.12 [0.10, 0.15]	<.001
**PIII (mV)**	0.07 [0.05, 0.10]	0.07 [0.04, 0.10]	0.07 [0.05, 0.11]	0.07 [0.05, 0.09]	0.621
**PaVR (mV)**	0.09 [0.04, 0.21]	0.05 [0.03, 0.13]	0.06 [0.03, 0.20]	0.09 [0.04, 0.33]	<.001
**PaVL (mV)**	0.04 [0.02, 0.05]	0.03 [0.02, 0.05]	0.04 [0.03, 0.06]	0.03 [0.02, 0.05]	0.200
**PaVF (mV)**	0.09 [0.05, 0.13]	0.07 [0.05, 0.10]	0.08 [0.06, 0.12]	0.08 [0.06, 0.11]	0.101
**PV1 (mV)**	0.09 [0.05, 0.14]	0.06 [0.03, 0.10]	0.06 [0.03, 0.10]	0.05 [0.03, 0.07]	0.020
**PV2 (mV)**	0.08 [0.05, 0.11]	0.06 [0.03, 0.10]	0.06 [0.04, 0.09]	0.05 [0.03, 0.07]	0.058
**PV3 (mV)**	0.08 [0.06, 0.11]	0.07 [0.04, 0.09]	0.08 [0.06, 0.10]	0.08 [0.07, 0.09]	<.001
**PV4 (mV)**	0.08 [0.05, 0.10]	0.06 [0.04, 0.08]	0.07 [0.06, 0.09]	0.08 [0.06, 0.09]	<.001
**PV5 (mV)**	0.07 [0.05, 0.09]	0.05 [0.04, 0.08]	0.07 [0.05, 0.09]	0.07 [0.06, 0.09]	<.001
**PV6 (mV)**	0.07 [0.05, 0.09]	0.05 [0.04, 0.08]	0.07 [0.05, 0.09]	0.07 [0.06, 0.08]	<.001

**RS ratio I**	12.3 [4.5, 26.0]	9.2 [2.8, 17.7]	14.1 [7.1, 25.1]	25.3 [10.0, 48.2]	<.001
**RS ratio II**	4.10 [1.19, 16.41]	2.76 [0.87, 10.09]	6.57 [1.68, 19.08]	20.5 [7.7, 42.4]	0.002
**RS ratio III**	0.69 [0.11, 4.01]	0.97 [0.17, 4.82]	0.89 [0.14, 4.07]	3.73 [0.36, 16.84]	0.543
**RS ratio aVR**	1.02 [0.20, 1.44]	1.05 [0.21, 2.06]	0.32 [0.10, 1.23]	0.29 [0.11, 1.19]	<.001
**RS ratio aVL**	9.45 [1.93, 23.64]	6.19 [1.88, 15.82]	12.10 [3.88, 23.38]	9.23 [1.68, 26.70]	0.003
**RS ratio aVF**	1.86 [0.32, 10.98]	1.12 [0.27, 6.86]	2.24 [0.45, 11.39]	10.5 [2.5, 29.8]	0.219
**RS ratio V1**	0.13 [0.08, 0.25]	0.17 [0.06, 0.68]	0.13 [0.06, 0.35]	0.18 [0.11, 0.29]	0.158
**RS ratio V2**	0.11 [0.06, 0.22]	0.12 [0.04, 0.38]	0.32 [0.12, 0.89]	0.37 [0.20, 0.66]	<.001
**RS ratio V3**	0.19 [0.07, 0.73]	0.13 [0.03, 0.53]	0.59 [0.19, 1.40]	1.19 [0.58, 2.80]	<.001
**RS ratio V4**	0.98 [0.23, 4.53]	0.66 [0.11, 2.15]	1.77 [0.79, 5.05]	4.83 [2.31, 16.90]	<.001
**RS ratio V5**	4.77 [1.36, 14.85]	2.17 [0.64, 9.03]	4.74 [1.70, 17.27]	16.9 [5.3, 48.1]	<.001
**RS ratio V6**	10.80 [3.12, 25.94]	5.56 [1.37, 13.87]	9.95 [3.47, 20.75]	34.5 [15.5, 67.9]	<.001
**TR ratio I**	0.15 [0.08, 0.27]	0.22 [0.11, 0.38]	0.20 [0.10, 0.35]	0.36 [0.25, 0.50]	0.181
**TR ratio II**	0.28 [0.16, 0.56]	0.34 [0.18, 0.76]	0.33 [0.15, 0.66]	0.41 [0.29, 0.56]	0.708
**TR ratio III**	0.76 [0.27, 1.80]	0.73 [0.29, 2.02]	0.82 [0.33, 2.26]	0.46 [0.21, 0.99]	0.539
**TR ratio aVR**	0.78 [0.44, 1.14]	0.74 [0.37, 1.33]	1.09 [0.54, 2.31]	0.87 [0.50, 2.40]	<.001
**TR ratio aVL**	0.17 [0.09, 0.33]	0.23 [0.11, 0.43]	0.20 [0.10, 0.40]	0.35 [0.19, 0.58]	0.085
**TR ratio aVF**	0.38 [0.21, 1.08]	0.50 [0.27, 1.33]	0.49 [0.21, 1.20]	0.39 [0.24, 0.74]	0.261
**TR ratio V1**	1.42 [0.94, 2.54]	1.86 [0.64, 3.73]	1.57 [0.72, 3.26]	0.91 [0.52, 1.58]	0.895
**TR ratio V2**	1.58 [0.96, 3.34]	2.47 [0.85, 7.00]	1.32 [0.50, 3.92]	1.12 [0.70, 1.93]	0.017
**TR ratio V3**	1.19 [0.46, 2.77]	1.83 [0.63, 6.78]	0.82 [0.43, 1.91]	0.64 [0.40, 0.97]	<.001
**TR ratio V4**	0.37 [0.17, 0.99]	0.68 [0.26, 2.67]	0.42 [0.19, 0.86]	0.37 [0.27, 0.51]	<.001
**TR ratio V5**	0.18 [0.10, 0.34]	0.33 [0.15, 0.67]	0.25 [0.15, 0.50]	0.32 [0.22, 0.43]	<.001
**TR ratio V6**	0.15 [0.10, 0.24]	0.20 [0.12, 0.40]	0.22 [0.12, 0.41]	0.31 [0.21, 0.40]	0.016
**RS slope (I)**	-0.02 [-0.03, -0.01]	-0.02 [-0.02, -0.01]	-0.03 [-0.04, -0.02]	-0.03 [-0.03, -0.02]	<.001
**RS slope (II)**	-0.02 [-0.03, -0.01]	-0.02 [-0.02, -0.01]	-0.03 [-0.05, -0.02]	-0.03 [-0.04, -0.02]	<.001
**RS slope (III)**	-0.02 [-0.03, -0.01]	-0.02 [-0.03, -0.01]	-0.02 [-0.04, -0.01]	-0.01 [-0.02, -0.01]	0.011
**RS slope (aVR)**	-0.01 [-0.01, -0.00]	-0.01 [-0.01, -0.00]	-0.02 [-0.03, -0.01]	-0.02 [-0.03, -0.00]	<.001
**RS slope (aVL)**	-0.02 [-0.02, -0.01]	-0.02 [-0.02, -0.01]	-0.02 [-0.03, -0.02]	-0.02 [-0.03, -0.01]	<.001
**RS slope (aVF)**	-0.02 [-0.02, -0.01]	-0.02 [-0.03, -0.01]	-0.02 [-0.04, -0.01]	-0.02 [-0.03, -0.01]	<.001
**RS slope (V1)**	-0.03 [-0.05, -0.02]	-0.03 [-0.04, -0.01]	-0.04 [-0.06, -0.03]	-0.04 [-0.05, -0.03]	<.001
**RS slope (V2)**	-0.06 [-0.08, -0.03]	-0.05 [-0.08, -0.02]	-0.06 [-0.09, -0.03]	-0.06 [-0.08, -0.04]	0.027
**RS slope (V3)**	-0.06 [-0.08, -0.04]	-0.05 [-0.08, -0.03]	-0.07 [-0.09, -0.04]	-0.06 [-0.08, -0.05]	0.006

**RS slope (V4)**	-0.05 [-0.08, -0.04]	-0.04 [-0.06, -0.02]	-0.07 [-0.10, -0.04]	-0.07 [-0.08, -0.05]	<.001
**RS slope (V5)**	-0.05 [-0.07, -0.03]	-0.03 [-0.04, -0.01]	-0.05 [-0.08, -0.03]	-0.05 [-0.06, -0.04]	<.001
**RS slope (V6)**	-0.03 [-0.05, -0.02]	-0.02 [-0.03, -0.01]	-0.03 [-0.05, -0.02]	-0.03 [-0.04, -0.02]	<.001
**ST slope I (mV/ms)**	0.00 [-0.00, 0.00]	0.00 [-0.00, 0.00]	0.00 [-0.00, 0.00]	0.00 [0.00, 0.00]	0.038
**ST slope II (mV/ms)**	0.00 [0.00, 0.00]	0.00 [-0.00, 0.00]	0.00 [0.00, 0.00]	0.00 [0.00, 0.00]	0.246
**ST slope III (mV/ms)**	0.00 [0.00, 0.01]	0.00 [0.00, 0.01]	0.00 [0.00, 0.01]	0.00 [-0.00, 0.00]	0.241
**ST slope aVR (mV/ms)**	0.00 [-0.00, 0.01]	0.00 [-0.00, 0.00]	0.00 [-0.00, 0.01]	0.00 [-0.00, 0.01]	<.001
**ST slope aVL (mV/ms)**	0.00 [-0.00, 0.00]	-0.00 [-0.00, 0.00]	-0.00 [-0.00, 0.00]	0.00 [0.00, 0.00]	0.246
**ST slope aVF (mV/ms)**	0.00 [0.00, 0.00]	0.00 [0.00, 0.01]	0.00 [0.00, 0.01]	0.00 [0.00, 0.00]	0.669
**ST slope V1 (mV/ms)**	0.01 [0.01, 0.02]	0.01 [0.00, 0.01]	0.01 [0.01, 0.02]	0.01 [0.01, 0.01]	<.001
**ST slope V2 (mV/ms)**	0.02 [0.01, 0.03]	0.01 [0.01, 0.02]	0.01 [0.01, 0.02]	0.01 [0.01, 0.02]	0.020
**ST slope V3 (mV/ms)**	0.02 [0.01, 0.03]	0.01 [0.01, 0.02]	0.01 [0.01, 0.02]	0.01 [0.00, 0.01]	0.005
**ST slope V4 (mV/ms)**	0.01 [0.00, 0.01]	0.01 [0.00, 0.01]	0.01 [0.00, 0.01]	0.00 [0.00, 0.01]	0.165
**ST slope V5 (mV/ms)**	0.00 [0.00, 0.01]	0.00 [0.00, 0.01]	0.00 [0.00, 0.01]	0.00 [0.00, 0.00]	0.209
**ST slope V6 (mV/ms)**	0.00 [-0.00, 0.00]	0.00 [-0.00, 0.00]	0.00 [-0.00, 0.00]	0.00 [0.00, 0.00]	0.040
**QRS area I (mV·ms)**	0.01 [0.01, 0.02]	0.01 [0.00, 0.02]	0.02 [0.01, 0.03]	0.01 [0.01, 0.02]	<.001
**QRS area II (mV·ms)**	0.01 [0.00, 0.01]	0.00 [-0.00, 0.01]	0.01 [0.00, 0.02]	0.01 [0.01, 0.02]	<.001
**QRS area III (mV·ms)**	-0.00 [-0.01, 0.00]	-0.00 [-0.01, 0.00]	-0.00 [-0.01, 0.00]	0.00 [-0.00, 0.01]	0.581
**QRS area aVR (mV·ms)**	-0.00 [-0.01, 0.00]	-0.00 [-0.01, 0.00]	-0.00 [-0.01, 0.00]	-0.00 [-0.00, -0.00]	<.001
**QRS area aVL (mV·ms)**	0.01 [0.00, 0.02]	0.01 [0.00, 0.02]	0.02 [0.01, 0.03]	0.01 [0.00, 0.01]	<.001
**QRS area aVF (mV·ms)**	0.00 [-0.00, 0.01]	-0.00 [-0.01, 0.01]	0.00 [-0.00, 0.01]	0.01 [0.00, 0.01]	0.358
**QRS area V1 (mV·ms)**	-0.01 [-0.02, -0.01]	-0.01 [-0.02, -0.01]	-0.01 [-0.02, -0.00]	-0.01 [-0.01, -0.00]	0.539
**QRS area V2 (mV·ms)**	-0.02 [-0.02, -0.01]	-0.01 [-0.03, -0.01]	-0.01 [-0.02, -0.00]	-0.01 [-0.01, -0.00]	<.001
**QRS area V3 (mV·ms)**	-0.01 [-0.02, -0.00]	-0.02 [-0.03, -0.01]	-0.01 [-0.02, 0.01]	0.00 [-0.01, 0.01]	<.001
**QRS area V4 (mV·ms)**	0.00 [-0.01, 0.02]	-0.00 [-0.01, 0.01]	0.01 [-0.00, 0.02]	0.01 [0.01, 0.02]	<.001
**QRS area V5 (mV·ms)**	0.02 [0.01, 0.03]	0.01 [-0.00, 0.02]	0.02 [0.00, 0.03]	0.02 [0.01, 0.02]	<.001
**QRS area V6 (mV·ms)**	0.02 [0.01, 0.03]	0.01 [0.00, 0.02]	0.02 [0.01, 0.03]	0.02 [0.01, 0.02]	0.003
**RR interval (ms)**	678.0 [594.7, 766.2]	772.5 [663.3, 905.4]	806.8 [700.4, 920.0]	810.0 [728.8, 909.0]	<.001
**RR interval SD (ms)**	19.7 [9.8, 54.1]	45.9 [16.5, 94.7]	32.2 [15.4, 73.4]	24.6 [14.3, 38.5]	<.001
**QRS duration (ms)**	100.0 [92.0, 116.0]	106.5 [94.0, 128.0]	92.0 [84.0, 104.0]	86.0 [80.0, 92.0]	<.001
**QT interval (segmented) (ms)**	389.4 [354.6, 421.4]	417.6 [377.6, 449.8]	421.1 [383.4, 452.8]	408.7 [386.5, 424.7]	<.001

**QT interval (ms)**	325.2 [305.1, 352.4]	341.1 [313.6, 364.0]	338.3 [314.8, 361.0]	320.0 [302.6, 333.8]	0.002
**QT interval SD (ms)**	25.5 [19.1, 34.9]	29.9 [22.8, 40.9]	25.2 [18.3, 33.9]	19.3 [14.7, 23.4]	<.001
**JT interval (ms)**	285.5 [250.6, 315.9]	314.8 [277.1, 344.3]	323.8 [292.2, 350.8]	313.9 [294.4, 329.9]	<.001
**T duration (ms)**	149.0 [130.2, 166.9]	166.2 [148.4, 180.0]	177.3 [160.4, 187.8]	186.6 [179.1, 190.9]	<.001
**T end (ms)**	646.0 [566.0, 861.0]	648.0 [582.5, 775.5]	626.0 [578.0, 693.0]	578.0 [552.0, 604.5]	0.065
**QRS onset (ms)**	224.0 [194.0, 502.0]	220.0 [198.0, 288.5]	206.0 [190.0, 240.0]	194.0 [180.0, 210.0]	0.013
**QRS end (ms)**	344.0 [296.0, 595.0]	342.0 [304.0, 434.0]	302.0 [280.0, 340.0]	282.0 [263.5, 300.0]	<.001
**S-peak to T-peak interval (ms)**	217.4 [201.5, 235.6]	228.5 [208.1, 254.7]	232.6 [211.7, 253.5]	219.4 [205.4, 233.4]	<.001
**S-peak to T onset (ms)**	126.3 [114.2, 147.1]	136.6 [117.6, 161.9]	137.9 [120.0, 159.6]	122.9 [110.0, 138.1]	0.004
**Q–S peak interval (ms)**	108.6 [100.9, 118.2]	111.1 [103.3, 122.8]	107.2 [94.9, 115.3]	97.9 [91.7, 106.0]	<.001
**Tpeak–Tend SD (ms)**	14.0 [9.9, 16.9]	13.5 [10.4, 18.0]	10.5 [7.1, 15.3]	8.75 [6.61, 11.21]	<.001
**QT dispersion (ms)**	106.0 [87.5, 130.2]	114.1 [90.0, 141.1]	104.8 [81.7, 136.2]	99.0 [80.7, 114.7]	0.004
**QRS dispersion (ms)**	80.5 [62.0, 95.6]	82.1 [67.1, 97.7]	75.9 [63.8, 95.4]	65.0 [55.2, 78.8]	0.010
**P duration (ms)**	91.3 [87.0, 93.7]	89.6 [84.5, 93.4]	91.1 [86.1, 93.7]	92.2 [89.2, 94.0]	0.256
**P end (ms)**	156.0 [140.0, 170.0]	150.0 [134.0, 166.0]	154.0 [140.0, 164.0]	148.0 [140.0, 158.0]	0.556
**R-wave duration (ms)**	83.6 [79.2, 86.6]	82.6 [77.7, 86.5]	80.3 [75.9, 84.4]	78.8 [75.7, 81.9]	0.002
**TP segment duration (ms)**	154.9 [124.2, 210.1]	215.6 [147.5, 321.6]	243.2 [168.2, 321.0]	268.7 [204.0, 348.5]	<.001
**Mean frequency I (Hz)**	6.24 [5.29, 7.44]	5.83 [4.49, 6.87]	6.39 [5.47, 7.45]	7.30 [6.48, 8.24]	<.001
**Mean frequency II (Hz)**	6.30 [4.54, 7.66]	5.61 [4.26, 6.78]	6.45 [5.05, 7.61]	7.21 [6.43, 8.01]	<.001
**Mean frequency III (Hz)**	5.85 [4.50, 7.42]	5.55 [4.20, 6.91]	6.10 [4.56, 7.36]	7.11 [5.75, 8.17]	0.037
**Mean frequency aVR (Hz)**	6.45 [5.20, 7.52]	5.77 [4.70, 6.76]	6.47 [5.64, 7.32]	7.42 [6.65, 8.15]	<.001
**Mean frequency aVL (Hz)**	6.12 [4.74, 7.39]	5.64 [4.33, 7.10]	6.24 [5.10, 7.36]	7.25 [6.05, 8.39]	<.001
**Mean frequency aVF (Hz)**	5.94 [4.25, 7.30]	5.40 [4.22, 6.95]	6.06 [4.23, 7.40]	7.04 [5.71, 7.95]	0.024
**Mean frequency V1 (Hz)**	5.86 [4.93, 6.61]	5.27 [4.51, 6.20]	6.21 [5.13, 7.10]	7.19 [6.44, 8.03]	<.001
**Mean frequency V2 (Hz)**	5.60 [4.72, 6.46]	5.00 [4.09, 5.87]	5.83 [4.59, 7.55]	6.53 [5.43, 7.74]	<.001
**Mean frequency V3 (Hz)**	5.84 [4.85, 6.92]	5.10 [4.13, 6.12]	6.33 [4.74, 7.92]	7.16 [6.14, 8.56]	<.001
**Mean frequency V4 (Hz)**	6.63 [5.18, 8.35]	5.56 [4.39, 6.87]	7.09 [5.53, 8.67]	8.13 [6.97, 9.06]	<.001

**Mean frequency V5 (Hz)**	6.98 [5.78, 7.88]	6.08 [4.43, 7.27]	7.11 [5.86, 8.36]	7.87 [7.01, 8.71]	<.001
**Mean frequency V6 (Hz)**	6.54 [5.67, 7.79]	5.65 [4.63, 6.90]	6.63 [5.51, 7.59]	7.58 [6.82, 8.27]	<.001
**Median frequency I (Hz)**	5.50 [4.50, 7.00]	5.00 [3.00, 6.00]	5.50 [4.00, 6.50]	6.00 [4.50, 7.00]	<.001
**Median frequency II (Hz)**	5.50 [3.50, 7.00]	4.50 [3.00, 6.00]	5.50 [3.00, 6.50]	6.00 [4.50, 7.00]	<.001
**Median frequency III (Hz)**	4.50 [3.00, 6.50]	4.00 [3.00, 6.00]	4.50 [3.00, 6.50]	6.00 [4.00, 7.00]	0.164
**Median frequency aVR (Hz)**	5.50 [4.00, 6.50]	5.00 [3.50, 6.00]	5.50 [4.50, 6.00]	6.50 [5.00, 7.00]	<.001
**Median frequency aVL (Hz)**	5.00 [3.50, 6.50]	4.50 [3.00, 6.00]	5.00 [3.50, 6.50]	6.00 [4.00, 7.12]	0.011
**Median frequency aVF (Hz)**	5.00 [3.00, 6.50]	4.00 [2.50, 6.00]	4.50 [2.50, 6.00]	6.00 [4.00, 7.00]	0.059
**Median frequency V1 (Hz)**	5.00 [3.50, 6.00]	4.00 [3.00, 5.50]	5.00 [4.00, 6.00]	6.50 [5.50, 7.00]	<.001
**Median frequency V2 (Hz)**	4.50 [3.50, 5.50]	3.50 [3.00, 4.50]	4.00 [2.50, 6.00]	4.50 [3.00, 7.00]	<.001
**Median frequency V3 (Hz)**	4.50 [3.50, 6.00]	3.50 [3.00, 5.00]	4.75 [3.00, 7.00]	5.75 [3.50, 8.00]	<.001
**Median frequency V4 (Hz)**	6.00 [3.50, 7.50]	4.00 [3.00, 6.00]	5.75 [3.50, 8.00]	7.00 [5.00, 8.00]	<.001
**Median frequency V5 (Hz)**	6.50 [5.00, 7.50]	5.00 [3.00, 6.50]	6.00 [4.00, 7.50]	7.00 [5.50, 7.50]	<.001
**Median frequency V6 (Hz)**	5.50 [5.00, 7.00]	4.50 [3.00, 6.00]	5.50 [4.00, 6.50]	6.50 [5.50, 7.12]	<.001
**Time-domain skewness (I)**	2.49 [1.37, 3.19]	2.12 [0.84, 3.09]	3.08 [1.87, 3.63]	2.70 [1.94, 3.35]	<.001
**Time-domain skewness (II)**	1.24 [-0.58, 2.58]	0.66 [-0.92, 2.28]	1.76 [0.44, 2.96]	2.36 [1.64, 3.16]	<.001
**Time-domain skewness (III)**	-1.46 [-2.69, 1.04]	-1.39 [-2.50, 0.90]	-1.64 [-3.13, 0.70]	0.29 [-2.11, 2.56]	0.209
**Time-domain skewness (aVR)**	-2.33 [-2.92, -0.94]	-1.64 [-2.68, -0.16]	-2.76 [-3.38, -1.73]	-2.67 [-3.26, -2.08]	<.001
**Time-domain skewness (aVL)**	2.36 [0.73, 3.16]	1.96 [0.11, 2.98]	2.87 [1.49, 3.69]	2.16 [0.39, 3.39]	<.001
**Time-domain skewness (aVF)**	-0.04 [-1.79, 1.94]	-0.51 [-1.94, 1.47]	0.55 [-1.45, 2.16]	1.90 [0.70, 3.16]	0.141
**Time-domain skewness (V1)**	-2.81 [-3.29, -2.14]	-2.52 [-3.07, -1.71]	-2.91 [-3.43, -2.13]	-3.43 [-3.93, -2.72]	0.004
**Time-domain skewness (V2)**	-2.75 [-3.20, -1.60]	-2.45 [-3.05, -1.62]	-1.92 [-2.89, -0.33]	-1.90 [-3.01, -0.65]	<.001
**Time-domain skewness (V3)**	-2.28 [-2.91, -0.85]	-2.41 [-3.10, -1.51]	-1.05 [-2.51, 0.47]	0.31 [-1.30, 1.51]	<.001
**Time-domain skewness (V4)**	-0.43 [-2.26, 2.34]	-1.40 [-2.52, 1.15]	0.96 [-0.98, 2.41]	2.37 [1.41, 3.19]	<.001
**Time-domain skewness (V5)**	2.40 [0.24, 3.31]	0.94 [-1.60, 2.86]	2.20 [0.62, 3.42]	3.06 [2.26, 3.62]	<.001
**Time-domain skewness (V6)**	2.81 [1.58, 3.37]	2.03 [0.24, 3.10]	2.74 [1.49, 3.53]	3.20 [2.57, 3.72]	<.001
**Time-domain kurtosis (I)**	7.75 [4.56, 11.31]	7.14 [4.08, 11.00]	10.94 [6.23, 14.44]	8.98 [5.12, 13.20]	<.001
**Time-domain kurtosis (II)**	5.36 [2.30, 8.74]	4.47 [1.98, 8.47]	7.02 [2.95, 10.97]	7.66 [4.08, 12.00]	<.001
**Time-domain kurtosis (III)**	6.80 [3.68, 11.65]	6.58 [3.33, 10.82]	8.73 [3.48, 14.24]	9.06 [4.45, 14.80]	0.018
**Time-domain kurtosis (aVR)**	7.16 [3.49, 10.23]	5.49 [2.53, 8.81]	9.47 [5.29, 13.33]	8.81 [5.36, 12.55]	<.001
**Time-domain kurtosis (aVL)**	7.63 [4.49, 11.35]	7.05 [3.81, 10.87]	10.55 [6.23, 15.50]	9.53 [4.95, 14.94]	<.001

**Time-domain kurtosis (aVF)**	5.43 [2.22, 9.22]	5.46 [2.57, 9.25]	6.53 [3.14, 11.30]	7.02 [2.90, 12.51]	0.021
**Time-domain kurtosis (V1)**	8.67 [5.81, 11.68]	7.52 [4.76, 10.68]	10.84 [7.54, 13.44]	13.4 [9.6, 16.8]	<.001
**Time-domain kurtosis (V2)**	8.11 [5.42, 11.20]	7.39 [4.36, 10.36]	7.81 [4.24, 11.51]	7.92 [4.31, 12.24]	0.556
**Time-domain kurtosis (V3)**	7.19 [4.39, 9.96]	7.31 [4.84, 10.92]	7.65 [3.80, 11.47]	5.91 [3.03, 9.61]	0.981
**Time-domain kurtosis (V4)**	7.23 [4.98, 10.23]	6.64 [4.26, 10.84]	7.70 [4.27, 12.52]	8.85 [4.94, 13.03]	0.011
**Time-domain kurtosis (V5)**	8.63 [5.34, 12.32]	7.24 [4.09, 11.44]	9.72 [5.14, 14.08]	11.5 [6.9, 15.3]	<.001
**Time-domain kurtosis (V6)**	9.38 [5.78, 12.45]	7.09 [4.08, 11.26]	10.00 [6.33, 14.07]	12.1 [8.2, 15.5]	<.001
**P axis (°)**	53.5 [34.8, 65.0]	54.0 [25.0, 67.0]	48.0 [31.5, 65.0]	54.0 [38.0, 63.0]	0.399
**QRS axis (°)**	3.00 [-28.00, 43.00]	-3.50 [-38.00, 45.00]	10.00 [-18.00, 34.00]	33.0 [13.0, 56.0]	0.413
**T axis (°)**	75.0 [41.0, 113.0]	85.0 [28.5, 123.8]	63.0 [16.5, 109.0]	39.0 [24.0, 55.2]	0.169
**QRS–T principal eigenvector angle (°)**	75.1 [27.2, 146.4]	73.8 [22.1, 141.8]	57.9 [22.2, 135.8]	25.5 [14.1, 38.8]	0.888
**Spatial peak QRS–T angle (°)**	155.1 [117.8, 167.9]	155.9 [126.2, 168.0]	141.4 [77.9, 164.9]	35.1 [20.6, 53.3]	0.014
**Spatial mean QRS–T angle (°)**	92.1 [53.6, 128.9]	97.8 [48.7, 127.9]	89.1 [54.2, 125.5]	91.5 [56.6, 121.2]	0.888
**Max QRS–T angle across planes (°)**	145.5 [92.6, 165.0]	151.5 [104.6, 168.4]	140.8 [97.1, 165.6]	137.9 [86.6, 163.0]	0.864
**QRS–T angle (frontal, °)**	99.0 [48.5, 151.1]	105.9 [52.9, 154.5]	90.9 [42.0, 140.5]	91.5 [45.3, 136.7]	0.557
**QRS–T angle (horizontal, °)**	93.6 [36.0, 140.7]	93.9 [35.4, 143.3]	89.7 [41.5, 131.5]	90.3 [41.8, 141.7]	1.000
**P max-to-mean vector ratio**	1.45 [1.29, 1.57]	1.44 [1.27, 1.62]	1.49 [1.35, 1.67]	1.58 [1.43, 1.80]	0.068
**Spatial mean P–QRS angle (°)**	89.1 [57.3, 107.4]	91.1 [58.3, 120.0]	86.7 [56.0, 116.4]	89.8 [62.7, 121.5]	0.648
**Spatial peak P–QRS angle (°)**	118.4 [73.2, 149.0]	106.4 [65.2, 142.1]	96.4 [41.8, 139.9]	148.3 [115.2, 160.4]	0.019
**T max-to-mean vector ratio**	1.48 [1.31, 1.74]	1.52 [1.38, 1.69]	1.67 [1.50, 1.88]	1.99 [1.83, 2.14]	<.001
**Ventricular gradient elevation (°)**	38.2 [14.4, 58.6]	41.0 [13.4, 63.2]	10.0 [-4.3, 36.3]	-1.96 [-10.88, 7.58]	<.001
**ln P-wave dipolar amplitude (ln mV)**	-1.31 [-1.70, -1.11]	-1.62 [-2.01, -1.31]	-1.46 [-1.75, -1.15]	-1.46 [-1.66, -1.23]	0.003
**Direction of max QRS (frontal, °)**	20.0 [2.1, 33.1]	19.7 [-10.5, 44.2]	24.0 [6.5, 33.7]	29.9 [21.2, 38.6]	0.706
**Direction of max QRS (sagittal, °)**	68.5 [42.1, 87.6]	78.1 [53.4, 92.3]	54.6 [22.3, 80.3]	17.5 [-2.8, 41.4]	<.001
**QRS azimuth (°)**	-42.0 [-127.3, 19.8]	-49.2 [-121.7, 15.2]	-68.9 [-134.8, 25.9]	-134.4 [-144.9, -102.2]	0.818
**Sine of VG azimuth (horizontal)**	0.68 [0.28, 0.91]	0.77 [0.28, 0.95]	0.23 [-0.09, 0.66]	-0.04 [-0.23, 0.15]	<.001

**Sine of VG azimuth (sagittal)**	0.86 [0.44, 0.97]	0.87 [0.44, 0.97]	0.31 [-0.18, 0.82]	-0.06 [-0.35, 0.28]	<.001
**Ventricular gradient (RVPO) (mV·ms)**	-2.26 [-8.70, 2.49]	1.36 [-4.27, 6.05]	-6.67 [-13.91, -1.23]	-14.9 [-19.5, -10.7]	<.001
**Max 3D QRS vector magnitude (mV)**	1.08 [0.72, 1.36]	0.85 [0.56, 1.15]	1.15 [0.83, 1.55]	0.86 [0.70, 1.07]	<.001
**Normalized P eigenvector-2 amplitude**	0.43 [0.31, 0.63]	0.40 [0.26, 0.57]	0.46 [0.29, 0.60]	0.47 [0.34, 0.63]	0.103
**Normalized QRS eigenvector-2 amplitude**	0.35 [0.21, 0.48]	0.33 [0.23, 0.48]	0.37 [0.25, 0.54]	0.37 [0.26, 0.53]	0.307
**Normalized T eigenvector-2 amplitude**	0.30 [0.20, 0.46]	0.31 [0.20, 0.48]	0.28 [0.18, 0.46]	0.18 [0.12, 0.29]	0.415
**Ratio of ln QRS to ln T non-dipolar components**	0.97 [0.71, 1.25]	0.95 [0.73, 1.23]	0.89 [0.72, 1.12]	0.73 [0.58, 0.92]	0.553
**ln P eigenvector-3 amplitude (ln mV)**	-4.05 [-4.62, -3.63]	-4.36 [-4.86, -3.96]	-4.26 [-4.80, -3.92]	-4.34 [-4.72, -3.98]	0.277
**ln QRS non-dipolar component (normalized)**	-1.25 [-1.54, -1.04]	-1.24 [-1.48, -1.01]	-1.20 [-1.49, -0.99]	-1.24 [-1.46, -1.01]	0.880
**ln T eigenvector-2 amplitude (ln mV)**	-1.75 [-2.34, -1.28]	-1.67 [-2.21, -1.25]	-1.39 [-1.94, -0.77]	-1.77 [-2.20, -1.33]	<.001
**ln T-wave dipolar amplitude (first eigenvector) (ln mV)**	-0.58 [-0.95, -0.16]	-0.53 [-0.90, -0.15]	-0.01 [-0.43, 0.33]	-0.04 [-0.34, 0.22]	<.001
**Mean T minus mean QRS area (mV·ms)**	51.2 [34.5, 72.2]	48.8 [33.6, 78.6]	54.0 [33.8, 81.6]	24.0 [17.3, 32.4]	0.097
**Mean T time–voltage area (mV·ms)**	21.6 [14.8, 29.6]	21.8 [15.4, 33.1]	29.2 [20.9, 42.8]	22.7 [18.0, 29.3]	<.001
**P area (right–left), sagittal (%)**	0.65 [0.07, 4.31]	0.76 [0.06, 5.24]	0.52 [0.03, 2.62]	0.32 [0.06, 1.22]	0.103
**P time–voltage area (mV·ms)**	6.44 [4.73, 8.60]	5.24 [3.76, 6.98]	5.71 [4.30, 7.88]	5.19 [4.24, 6.45]	0.085
**QRS area (right–left), sagittal (%)**	0.61 [0.20, 2.22]	0.42 [0.05, 2.52]	0.27 [0.04, 1.39]	0.56 [0.12, 1.54]	0.024
**Ventricular gradient minus T area (mV·ms)**	33.9 [24.3, 47.6]	31.2 [21.8, 47.5]	32.1 [22.4, 48.7]	19.4 [14.1, 25.0]	0.781
**P axis azimuth (°)**	11.23 [-85.67, 102.55]	-4.73 [-89.94, 82.77]	13.32 [-88.90, 106.08]	2.02 [-80.24, 92.02]	0.116
**P axis elevation (°)**	1.82 [-20.24, 24.25]	0.00 [-26.73, 22.37]	0.00 [-18.31, 18.67]	0.00 [-13.94, 15.79]	0.690
**Q-wave amplitude (Lead Z) (mV)**	-0.01 [-0.03, 0.01]	-0.00 [-0.02, 0.02]	0.02 [-0.00, 0.03]	0.02 [0.01, 0.04]	<.001
**QTc (segmented) (ms)**	436.8 [418.5, 461.3]	451.3 [432.7, 469.1]	450.5 [431.9, 469.8]	437.1 [424.8, 449.7]	<.001

**R-wave amplitude (Lead Y) (mV)**	0.22 [0.02, 0.42]	0.11 [-0.05, 0.29]	0.30 [0.05, 0.60]	0.38 [0.25, 0.56]	<.001
**Sine of max T angle (horizontal)**	-0.56 [-0.83, -0.06]	-0.69 [-0.91, -0.04]	-0.22 [-0.67, -0.00]	-0.02 [-0.20, 0.01]	<.001
**Sine of max T angle (sagittal)**	-0.82 [-0.96, -0.17]	-0.81 [-0.96, -0.22]	-0.30 [-0.82, -0.00]	-0.03 [-0.33, 0.01]	<.001
**Spatial ventricular activation time (ms)**	78.0 [64.0, 94.0]	84.0 [64.5, 104.0]	74.0 [60.0, 90.5]	64.0 [54.0, 76.0]	0.002
**T index (unitless)**	2.24 [1.56, 3.51]	1.64 [1.19, 2.59]	1.27 [1.09, 2.04]	1.13 [1.06, 1.34]	<.001
**Tpeak–Tend (ms)**	60.2 [43.3, 75.1]	76.9 [59.0, 86.8]	84.3 [67.4, 92.3]	90.5 [84.6, 93.7]	<.001
**PR Duration**	71.5 [52.3, 90.3]	81.1 [64.5, 106.0]	74.1 [57.4, 93.9]	60.3 [48.5, 75.6]	<.001
**Abnormal ECG**	45.4% (64)	71.6% (179)	61.1% (127)	0.0% (0)	
**Borderline ECG**	12.1% (17)	4.4% (11)	17.8% (37)	0.0% (0)	
**Sinus bradycardia**	5.0% (7)	7.2% (18)	10.1% (21)	0.0% (0)	
**Sinus Rhythm**	56.0% (79)	64.4% (161)	71.2% (148)	100.0% (500)	
**Sinus Tachycardia**	29.8% (42)	12.8% (32)	11.1% (23)	0.0% (0)	
**Low Limb Voltage**	7.1% (10)	12.4% (31)	1.9% (4)	0.0% (0)	
**Low Precordial Voltage**	4.3% (6)	10.8% (27)	4.3% (9)	0.0% (0)	
**Left Ventricular Hypertrophy**	29.1% (41)	22.8% (57)	35.1% (73)	0.0% (0)	
**Left Axis Deviation**	25.5% (36)	31.2% (78)	17.8% (37)	100.0% (500)	
**Left Atrial Enlargement**	17.0% (24)	7.6% (19)	4.3% (9)	0.0% (0)	
**ST-T Changes**	22.0% (42)	38.0% (65)	41.7% (83)	0.0% (0)	
**T Wave Changes**	15.6% (22)	18.8% (47)	13.9% (29)	0.0% (0)	
